# Valsalva Retinopathy Masking as a Retinal Detachment on Point-of-care Ocular Ultrasound: A Case Report

**DOI:** 10.5811/cpcem.2022.1.55173

**Published:** 2022-03-07

**Authors:** Steven Johnson, Thomas Ryan, Amro Omari, Samantha Schneider, Amit Bahl

**Affiliations:** * Beaumont Hospital, Department of Emergency Medicine, Royal Oak, Michigan; † Beaumont Hospital, Department of Ophthalmology, Royal Oak, Michigan; ‡ Ascension Eye Institute, Department of Ophthalmology, Warren, Michigan

**Keywords:** ocular ultrasound, point-of-care ultrasound, ophthalmology, case report, pre-retinal hemorrhage, valsalva retinopathy

## Abstract

**Introduction:**

Approximately two million people present to the emergency department (ED) with eye-related complaints each year in the United States. Differentiating pathologies that need urgent consultation from those that do not is imperative. For some physicians, ocular ultrasound has eclipsed the dilated fundoscopic exam as the standard posterior segment evaluation in the ED.

**Case report:**

A 60-year-old female presented with sudden onset visual disturbance in her right eye. Point-of-care ultrasound showed a hyperechoic band in the posterior segment concerning for a retinal detachment. Ophthalmology was consulted and diagnosed the patient with a condition known as Valsalva retinopathy. The patient was discharged from the ED with expectant management.

**Conclusion:**

This case highlights an important differential diagnosis that should be considered when ocular ultrasound demonstrates a hyperechoic band in the posterior segment. While previous literature has demonstrated that emergency physicians are able to accurately identify posterior segment pathology using ultrasound, there is limited information regarding their ability to differentiate between pathologies, some of which may not require urgent consultation. We highlight the important differentials that should be considered when identifying posterior segment pathology on point-of-care ultrasound and their appropriate dispositions.

## INTRODUCTION

Approximately two million people present to the emergency department (ED) with eye-related complaints each year in the United States.^1^ Point-of-care ultrasound (POCUS) is a safe and effective way to screen for and triage intraocular pathologies, especially those of the posterior segment such as retinal detachments (RD). Prior research has shown that emergency physicians can perform ocular POCUS with high diagnostic accuracy.^2^ Valsalva retinopathy (VR) is an acute pathology of the posterior segment that can be identified using POCUS. However, POCUS findings and the clinical presentation of VR may be difficult to discern from RD. Although VR can cause vision loss that may need ophthalmic intervention, unlike RD, VR does not need immediate evaluation by a vitreoretinal surgeon.^3^ Thus, differentiating these pathologies can make a significant impact on patient care. Here we describe a case of Valsalva retinopathy that was successfully differentiated from a RD by careful ultrasonography.

## CASE REPORT

**Image 1:**
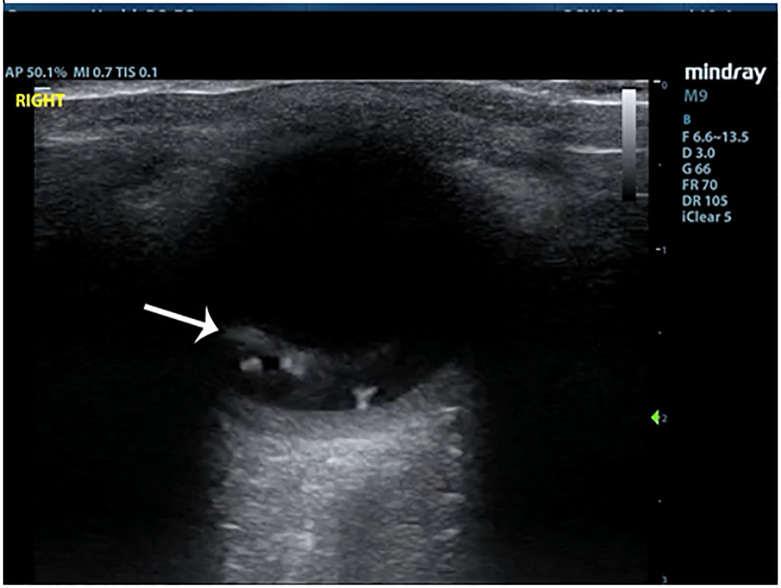
B-mode ultrasound of the right eye showing hyperechoic band (arrow) in the posterior segment with underlying scattered echoes.

**Image 2:**
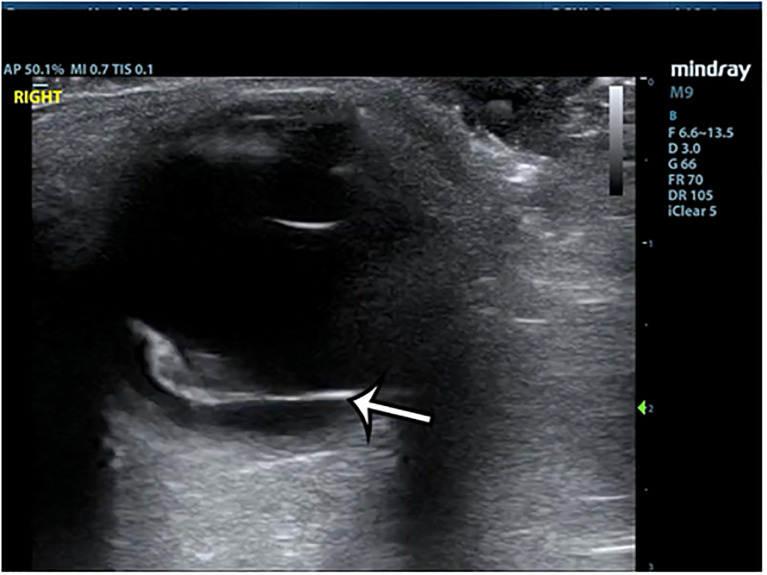
B-mode ultrasound of the right eye showing hyperechoic band (arrow) in the posterior segment spanning the entire globe.

A 60-year-old female with a past medical history of hypertension and type 2 diabetes presented to the ED due to vision changes in the right eye. She noted that after a sneezing fit, she experienced a sudden onset of floaters and a large cloud coming down into her vision. She described it as if she were looking through blood or ink spots. She did not have any eye pain and denied any trauma to the eye. She had no prior history of ophthalmic surgery or any anticoagulant use. Visual acuity was 20/30 in the left eye and 20/70 in the right eye. On exam, pupils were equal, round, and reactive, extraocular movements were intact, and the anterior segment exam was unremarkable. Confrontational visual fields were intact. The emergency physician performed an ultrasound of the right eye, and the images were obtained (Images 1 and 2, Video).

The POCUS showed a hyperechoic line raised off the posterior segment of the eye, which crossed the boundary of the optic nerve sheath. It was minimally mobile with eye movement, and scattered echoes were seen behind this stripe suggesting hemorrhage. Ophthalmology was consulted and observed extensive vitreous, intraretinal, and subhyaloid hemorrhage. The retina was not corrugated and there were no retinal breaks on their dilated eye exam, although some of the view was obscured by vitreous hemorrhage.

**CPC-EM Capsule**

What do we already know about this clinical entity?

*Valsalva retinopathy is a common cause of pre-retinal hemorrhage and is not acutely vision threatening but may be confused with other more serious pathologies on Point-of-care ultrasound (POCUS)*.

What makes this presentation of disease reportable?

*In our case ocular POCUS showed a hyperechoic line raised structure off the posterior segment, which crossed the boundary of the optic nerve, representing the pre-retinal membrane rather than the retina*.

What is the major learning point?

*Point-of-care ultrasound can differentiate pre-retinal hemorrhage from other pathologies that cause permanent vision loss by using several key features: echogenicity, mobility, and relation to the optic nerve*.

How might this improve emergency medicine practice?

*Since pre-retinal hemorrhage is not acutely vision threatening, accurate diagnosis by the ED physician may help alleviate the cost of unnecessary emergent consults and transfers in the future*.

The patient returned to retina clinic the next morning for follow-up assessment, where repeat ultrasound was performed. This study confirmed that the retina was flat and that there were no signs of a retinal break underlying the hemorrhage. Based on the exam findings of retinal hemorrhages in multiple layers and recent sneezing history, along with an intact retina on ultrasound, the diagnosis of Valsalva retinopathy was made. No immediate intervention was required, but the patient eventually underwent a vitrectomy to remove the large amount of vitreous hemorrhage.

## DISCUSSION

Point-of-care ultrasound is commonly used in the ED by both ophthalmologists and emergency physicians to aid in the diagnosis of acute posterior segment pathologies that can cause permanent visual impairment. In this patient, the predominant findings were vitreous opacities and a raised hyperechoic strip that was concerning for a RD. Emergency physicians should be aware that these findings have several additional differential diagnoses that should be considered. There are distinctive features that can be observed on POCUS to help differentiate between these entities (Table).

Retinal detachments are relatively common, occurring at an incidence of 12.5 per 100,000.^4^ On ultrasound it takes the appearance of a linear hyperechoic structure raised off the back of the eye. It does not cross the optic nerve and is mobile with extraocular movements. One end will be adherent to the edge of the optic nerve sheath, but the other ends may or may not be free. Spontaneous posterior vitreous detachment (PVD) is an even more commonly seen posterior segment pathology. Posterior vitreous detachment occurs in about 24% of patients aged 50–59 and 87% of patients over 80 years old.^5^ A PVD will generally require higher gain to be visible and will move with a “swishing” motion with extraocular movement. A less hyperechoic band may be seen posteriorly and will appear very thin compared to the thicker, very hyperechoic band seen in a RD. In a PVD this band represents the posterior hyaloid of the vitreous and not the retina; therefore, it can cross the optic nerve. It is notable that a VD and RD often occur together, and care must be taken not to miss a RD in cases of vitreous pathology. These are the most common posterior segment pathologies seen on POCUS, but as this case demonstrates there are other differential diagnoses that have similar findings.

The ultrasound findings on this patient were consistent with pre-retinal hemorrhage. In cases of pre-retinal hemorrhage, retinal capillaries rupture, causing blood to accumulate either in the level of the subhyaloid space, between the retina and the posterior vitreous face, or under the inner limiting membrane (ILM). If there is no hemorrhage overlying the macula, the patient may be completely asymptomatic. In this case, the patient’s pre-retinal hemorrhage was likely caused by her sneezing fit, suggesting a diagnosis of Valsalva retinopathy. The linear hyperechoic structure seen in this case accumulated above the retina, while the retina itself remained fully attached. The echogenic material seen behind the ILM is pre-retinal blood. With extraocular movement, there may be some movement seen, but it won’t move in the swishing/swirling pattern that a PVD will. Additionally, given that this hyperechoic band is not the retina, it can cross the optic nerve, as was seen in this case.

Another entity that should be considered is choroidal detachment. The choroid, which sits behind the retina, can detach from the sclera, and appear similar to the above pathologies. However, the detachments are deeper on ultrasound and appear convex (Table). While this can occur spontaneously, this less common entity usually occurs in the setting of hypotony after intraocular surgery. Emergency physicians should maintain a high index of suspicion in at-risk patients, and early ophthalmology consultation is recommended in these cases.

## CONCLUSION

Valsalva retinopathy, a common cause of pre-retinal hemorrhage, has some characteristic features on POCUS. As was found in our case, POCUS can help to differentiate it from other pathologies that cause permanent vision loss. Given that pre-retinal hemorrhage is not acutely vision threatening, accurate diagnosis by the emergency physician may help alleviate the cost of unnecessary emergent consults and transfers in the future. However, while current literature demonstrates that emergency physicians can accurately identify RDs and PVDs on POCUS, more studies are needed to assess whether ED-performed POCUS can safely exclude RD in cases of pre-retinal hemorrhage.

## Supplementary Information

Video A narrated B-mode ultrasound video of the right eye showing a thin, minimally mobile, hyperechoic band in the posterior segment that crosses the optic nerve, with underlying scattered echoes.

## Figures and Tables

**Table:** Summary of ultrasound findings of posterior chamber pathology.

Pathology	Movement	Morphology/ Prominence	Respects optic nerve boundary?
Retinal detachment	Moderate movement, may be free on one end	Visible at low gain	Yes
Vitreous detachment	Moves freely in swishing/ swirling motion	Visible at high gain	No
Pre-retinal hemorrhage	Little to none	Thin, hyperechoic strip with echogenic material posterior	No
Choroidal detachment	Little to none	Thick and convex	Yes
